# A Miniaturised Protocol for Feeding Measurements in Daphnids

**DOI:** 10.3390/mps9040102

**Published:** 2026-07-01

**Authors:** Izabela Antepowicz, Antonia Despotidi, Emma Rowan, Mbuyiselwa Shadrack Moloi, Silke Aulhorn, Harry Esmonde, Konstantinos Grintzalis, Eberhard Küster

**Affiliations:** 1 School of Biotechnology, Dublin City University, D09 Y5NO Dublin, Ireland; izabela.antepowicz3@mail.dcu.ie (I.A.); antonia.despotidi2@mail.dcu.ie (A.D.); emma.rowan4@mail.dcu.ie (E.R.); 2 UFZ Helmholtz Center for Environmental Research, 04318 Leipzig, Germany; mbuyiselwa.moloi@ufz.de (M.S.M.); silke.aulhorn@ufz.de (S.A.); 3 School of Mechanical and Manufacturing Engineering, Dublin City University, D09 Y5NO Dublin, Ireland; harold.esmonde@dcu.ie

**Keywords:** *Daphnia magna*, phenotypic endpoints, pollution monitoring

## Abstract

Daphnids, commonly known as water fleas, are freshwater planktonic microcrustacean species used as model organisms in ecotoxicology, particularly in regulatory frameworks that adhere to OECD and ISO standards. Mortality is the most common endpoint in toxicity testing; however, more sensitive indicators are required to assess sublethal acute effects of pollutants. The use of feeding impairment as a toxicity phenotypic endpoint in daphnids is considered a cost-effective approach that aligns with the 3Rs principle (Replace, Reduce, Refine) and is more physiologically and environmentally relevant. Current feeding methods are inefficient due to the large test volumes and extended incubation periods required. In this paper, we present a miniaturised protocol to assess feeding behaviour following exposure to chemicals in daphnids. The method is based on the consumption of algae, which is measured with chlorophyll fluorescence. The optimised protocol is more robust and rapid, and results can be obtained in 30 min and in a 96-well plate. Responses in feeding rate were investigated using this miniaturised protocol following exposure to a range of prevalent pollutants, which include two metals and, as a more realistic sample, a leachate from smoked cigarette filters. All three pollutants were tested at sublethal concentrations. This method provides an efficient approach to assess the toxicity of chemicals and water quality.

## 1. Introduction

Traditional aquatic pollution monitoring relies heavily on chemical analysis and the quantification of pollutants in water samples collected from aqueous environments [1,2]. However, the limited biological relevance and mechanistic understanding afforded by these analytical techniques has driven the adaptation of New Approach Methodologies (or NAMs), which provide sensitive endpoints for robust risk assessment [3]. Daphnids are ubiquitous in freshwater systems and are routinely integrated into the context of NAMs in ecotoxicological testing [4]. Responses of daphnids to toxicant exposure are frequently assessed using non-invasive phenotypic endpoints. Attributed to their characteristic filter feeding behaviour, daphnids clear the water from microorganisms and food-bound xenobiotics and serve as a food source for higher trophic level organisms. The vast majority of acute ecotoxicity assessments in daphnids predominantly focus on mortality and immobilisation following the OECD guideline 202 [5], while sublethal effects that require more sensitive endpoints remain largely underexplored [6].

Feeding inhibition in daphnids as a toxicity endpoint is believed to fill a gap as it improves the ecological relevance of toxicity testing and markedly reduces the duration of the assessments, thus allowing the effects of chemicals and pollutants to be detected within hours rather than weeks, which is necessary for survival or fecundity endpoints [7]. Feeding rate serves as a physiologically relevant endpoint of an individual response and represents the primary source of energy acquisition in animals and is therefore closely associated with performance, growth and reproduction [8]. Chemicals that inhibit grazing activity in *Daphnia* could lead to harmful effects on higher trophic organisms that extend to both population dynamics and community structure within natural ecosystems [9]. From the first paper of Gophen and Geller (1984) [10] to a more recent paper by Wang and Wang (2023) [11], who showed that daphnids selectively ingested larger and positively charged plastics, there has been great discussion about feeding in daphnids. Fluorescent microparticles can be used as “measurable food” to assess the impact of chemicals on the grazing activity of daphnids; however, in contrast to algal cells, the use of polymeric microparticles generates chemical waste (microplastics). The lack of environmental relevance is a major limiting factor, and thus using green microalgae as the food source to investigate feeding rates can be a better alternative [7]. Additionally, *Daphnia* showed a preference for algae particles over others [12]. The assessment of feeding behaviour using algal cells is based on the consumption of microalgae over a fixed time. Absorbance, chlorophyll fluorescence and microalgal labelling can be used to quantify the consumed (ingested) algae. Current algal feeding assays typically require large test volumes and long incubation periods, ranging from 4 to 24 h [13,14,15,16]. When test times are prolonged, algal cells may sink to the bottom of the test vessel, potentially confounding the measurements. It is worth mentioning the importance of feeding as a phenotypic endpoint, and our group has extensively studied feeding behaviour and developed fast performing assays [16,17,18,19].

In this study, we optimised a miniaturised feeding assay which is based on the consumption of algae. The optimised protocol was used to investigate the responses of feeding rate following exposure to several widely encountered pollutants at non-lethal concentrations. There are several advantages to our optimised protocol, including robustness; the results can be generated fast, in 30 min and in a miniaturised setting, facilitating high-throughput analysis of many samples.

## 2. Experimental Design

**Figure 1:**
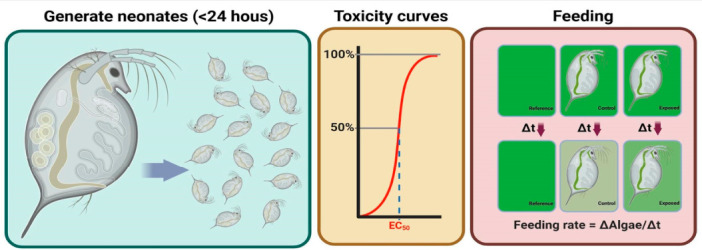
General outline of the steps of the multiparametric protocol.

The procedure of the protocol (outlined in Figure 1) begins with the exposure of neonates (<24 h) to the pollutants or chemicals of interest. Typically, acute exposures in neonates are for 24 or 48 h (steps 1–3), mimicking OECD TG 202. Subsequently, neonates are collected and washed of any remains of chemicals/pollutants in clear media and then split into groups in wells (steps 4–6). The separation of exposure and feeding were based on previous methods and additionally avoided any implications of the pollutants on the feeding test (e.g., binding of positively charged metals to the negatively charged algae surface, thus possibly increasing intoxication via additional uptake of the substance via food and not only via the water phase). An algal suspension was freshly prepared fresh (steps 7–8), and a 96- or 48-well plate was the preferred format for the feeding experiment (steps 4–6). To account for the available algal food during the test, wells without daphnids were also used. Finally, the algae suspension was added, and the samples were incubated to feed for 30 min (step 7). At the end of feeding incubation, the wells were mixed before sampling algae, and the wells were sampled and fluorescence measured (steps 8–9).

### 2.1. Materials

Salts for *Daphnia* culture media (ADaM, Klüttgen et al. 1994 [20]): Synthetic sea salt (0.333 g/L), CaCl_2_·2H_2_O (117.6 g/L), MgSO_4_·7H_2_O, NaHCO_3_ (25.2 g/L) and SeO_2_ (1.4 g/L).

Algae: In this study, algae of the species *Scenedesmus vacuolatus* were used as food for the daphnids.

Pollutants tested: As examples of common pollutants, two substances (dissolved in ADaM medium) were used: CdCl_2_*H_2_O (99% purity, CAS RN 35658-65-2, Merck KGaA, Darmstadt, Germany) and ZnSO_4_*7H_2_O (p.a., CAS RN 7446-20-0, Sigma Aldrich, St. Louis, MO, USA). The selection of these metals was based on previous studies, and we expected that they would affect the feeding rate, causing a decrease. In addition, a leachate from smoked cigarette filters was tested as a complex mixture which includes a variety of chemicals; thus, it would reflect a richer chemical composition. One smoked filter was leached for 24 h under shaking (125 rpm, Edmund Bühler shaker KL-2, Hechingen, Germany) at room temperature in ADaM medium. The leachate was cleared by filtration (0.22 μm).

### 2.2. Equipment

- Microplate reader (SpectraMax ID3, Molecular Devices, San Jose, CA, USA);
- 48- and 96-well plates (clear plates for the feeding test and black well plates for the fluorescence measurement);
- Counter for algae (Casy 1, Model TTC, Schärfe System) or haemocytometer.

## 3. Procedure

### 3.1. Exposure to Chemicals to Assess Toxicity (30 min for Setup and 24 or 48 h for Exposure to Chemicals)

Collect neonates (<24 h) from the third brood of their mothers in clean media.

Expose fifteen neonates in 50 ml media at different concentrations of pollutants. Incubate for 24 or 48 h in controlled light and temperature conditions (16 h:8 h of light:dark photoperiod and 20 °C). Different or higher densities of neonates/volume might also be used, but one would need to adapt to the same density in the feeding assay exposure (see point 5 below).


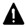
 **CRITICAL STEP** For each replicate of any concentration, a minimum of four replicates with fifteen animals each is advised for best results.

Plot the toxicity curves using the Hill model and decide the working non-lethal concentrations for exposures (~EC_20_ of immobilisation as the highest starting test concentration).

### 3.2. Exposure to Chemicals (30 min to Setup and 24 or 48 h for Exposure to Chemicals)

Collect neonates (<24 h) from the third brood of their mothers in clean media.

Expose 120 neonates in 400 mL media at the selected non-lethal concentrations for pollutants. Incubate for 24 or 48 h in controlled light and temperature conditions (16 h:8 h of light:dark photoperiod and 20 °C). As written above, densities can be increased if needed [18,21].


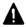
 **CRITICAL STEP** This part can be scaled accordingly for the number of animals required for the feeding; however, for each concentration tested for the feeding assay, a minimum of eight to ten replicates is proposed.

Collect all alive animals in clean media to remove any chemical remains. Animals can be separated from the media using suitable sieves (e.g., cell strainer with 100 µm mesh size, Labsolute, Th. Geyer, Renningen, Germany).

### 3.3. Preparation of Algae Suspension for Feeding and Standard Curve for the Correlation of Algae Fluorescence and Counts (20 min)

The aim was to create a fresh algal suspension of a fixed concentration of cells which will be used as the food for daphnids. The optimised range of concentration of *S. vacuolatus* algae is between 125,000 and 175,000 cells/mL in the feeding incubation; however, depending on algae species this might need adjustment (see troubleshooting table). Since in the assay the animals are added in half the volume (i.e., 150 μL or 0.5 mL), the concentration for the algae stock should be double that required for the feeding incubation. The concentration of algae can be measured with a haemocytometer or with a cell counter (the CASY counter was used in this study) in different dilutions, and fluorescence was measured in 200 μL volumes in a black 96-well plate. A standard curve was prepared for algae concentration in cells/mL (x axis) and relative fluorescence units-RFU (y axis), which can be used to convert the fluorescence measurements to actual algae cells. A Supplementary Excel file is provided for all calculations with worked out examples.


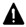
 **CRITICAL STEP** Once the algae stock has been prepared, it should be kept under continuous agitation with a magnet and stirrer before pipetting the algae into the wells. This is important to ensure that the concentration of algae is constant and reproducible in every well. Although in other methods algae are incubated for longer periods and they are expected to settle, mixing the stock continuously before pipetting ensures accuracy in each well for the loading of the same amount of algae. Additionally, during the short incubation period for feeding, no mixing is required as daphnids will mix while they swim in the media. Finally, we also observed that fluorescence and algae cells do not alter during this short incubation for feeding, and the method is robust and reproducible.

### 3.4. Incubation for Feeding (30 min for Preparation and 30 min for Incubation)

The protocol was developed in two versions, with eight and ten animals in 300 μL and 1 mL, respectively. At minimum, eight to ten replicates per condition is recommended. The main aim is to ensure reproducibility, and a consumption of algae for approximately 50% to 80% in the unexposed control to ensure any changes in feeding due to chemical exposure (usually observed as decrease in feeding) can be accurately monitored.

#### 3.4.1. Feeding Assay in 48-Well Plates (1 mL Version)

Transfer ten individuals in 500 μL of daphnid media in wells of a 48-well plate. Use wells without daphnids as well, with 500 μL of daphnid media only, which can be used to calculate the difference in fluorescence.

Add 500 μL from the algae suspension and incubate for 30 min.


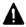
 **CRITICAL STEP** After incubation, mix the content of each well with a pipette to ensure homogeneity in the sample take. Then, transfer 200 μL from the suspension to a black well to measure fluorescence at Ex/Em 485/665 nm.

Calculate the difference in fluorescence from wells without daphnids (FU_wells without daphnids_ − FU_wells with daphnids_) and convert the difference in fluorescence to the amount of ingested algae (F) using the working out Excel file.

Finally, calculate the % inhibition in feeding (%IF) from each condition of chemical exposure in comparison to the unexposed control, as follows:

%IF_chemical_ = 100 (F_unexposed daphnids_ − FU_chemical exposed daphnids_)/F_unexposed daphnids_.

#### 3.4.2. Feeding Assay in 96-Well Plates (300 μL Version)

Transfer eight individuals in 150 μL of daphnid media in wells of a 96-well plate. Use wells without daphnids as well, with 150 μL of daphnid media only, which will be used to calculate the difference in fluorescence.

Add 150 μL from the algae suspension and incubate for 30 min.


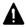
 **CRITICAL STEP** After incubation, mix the content of each well with a pipette to ensure homogeneity in the sample take. Then, transfer 200 μL from the suspension to a black well to measure fluorescence at Ex/Em 485/665 nm.

Calculate the difference in fluorescence from wells without daphnids (FU_wells without daphnids_ − FU_wells with daphnids_) and convert the difference in fluorescence to the amount of ingested algae (F) using the Excel file.

Finally, calculate the % inhibition in feeding (%IF) from each chemical in comparison to the unexposed control as follows:

%IF_chemical_ = 100 (FU_chemical exposed daphnids_ − F_unexposed daphnids_)/F_unexposed daphnids_.

### 3.5. Troubleshooting

The protocol presented is straightforward with a minimum number of steps. A troubleshooting table (Table 1) is added for possible caveats.

## 4. Expected Results

The protocol was optimised and can be applied as a fast and robust approach to assess the impact of any chemical on the physiology of daphnids. Physiological endpoints offer greater sensitivity for detecting sublethal effects caused by various stressors, as changes can be observed at lower toxicant concentrations and before immobilisation or death occurs [22]. Feeding assays are commonly employed in toxicity testing in daphnids [13,23]. In a study by Rodrigues et al. (2025) [24], the feeding assay was conducted based on the ingestion of microalgae in 6-well microplates with 12.5 mL and absorbance was measured from the consumption of five *D. magna* (4 to 5 days old) in the darkness for 24 h. The assay was carried out in the absence of light to prevent algal growth. Furthermore, sufficient sensitivity in conventional setups was achieved by using adult *Daphnia*, typically four or five days old [25]. Similar setups were used in other studies [26,27,28].

**Figure 2:**
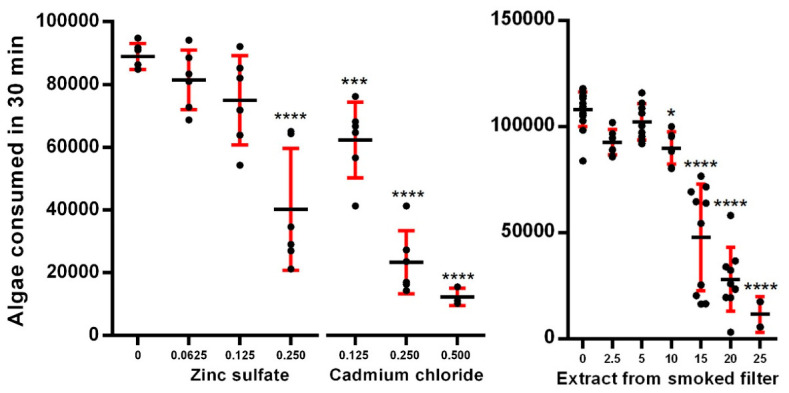
The impact of pollutants on feeding of daphnids. Daphnids were exposed to CdCl_2_, ZnSO_4_ (in mg/L) or an extract from smoked cigarette filters (in mL extract/50 mL) for 24 h, and feeding was subsequently assessed. Using the calculated values from the Supplementary Excel files (all data provided), dose-dependent differences in feeding rates were identified. For metal, data represent six measurements per condition tested; however, for the smoked extract we experienced mortality as concentration increased. Statistically significant changes were identified by one-way ANOVA, followed by Dunnett’s post hoc test, from the unexposed control (* *p* < 0.05, ** *p* < 0.01, *** *p* < 0.001, **** *p* < 0.0001).

In the present study, the feeding rate allowed us to observe sublethal toxic actions of chemicals in 30 min after 24 h of exposure of neonates using the 300 μL version in 96-well plates. Multiple replicates containing algae and no animals were performed in parallel to the assay to account for potential algae growth during incubation. Neonates ingest significantly fewer algae than adult *Daphnia* because of their smaller body size. By miniaturising the assay, chemical consumption and test volumes are reduced and laboratory space requirements are minimised. Collectively, this enables a higher throughput of samples and considerable time and cost savings [18]. In this study we provide results from the analysis of a number of pollutants with the optimised method for 300 μL (Figure 2). Additionally, we provide a worked out indicative exemplar of our outlined approach as a Supplementary Excel file with automated calculations to assist the user. The technical reproducibility of the method presented is reflected in the low coefficient of variance (which is usually below 10%, with the exception of a higher concentration where effects are more severe, as seen in the Excel provided) from independent replicates of each sample, which is a direct result of the simplicity of the assay performed with a minimum number of steps.

Feeding was clearly decreased following exposure to metals or the smoked cigarette filter leachate. Specifically, for zinc sulphate there was a trend of a decrease of 8%, starting from the lowest concentration (0.0625 mg/L), to 16% for the middle (0.125 mg/L) and a significant decrease of 55% in the highest (0.25 mg/L) concentration. For cadmium chloride, all three concentrations tested significantly decreased feeding, by 30%, 74% and 86%, respectively. For the smoked filter leachate feeding, a decrease followed a concentration dependence, with 17% in the 10 mL/50 mL, followed by 55% in the 15 mL/50 mL and 74% and 89% for the 20 and 25 mL/50 mL, respectively. However, at the highest concentrations for cadmium chloride and for the leachate we observed death; therefore, the number of replicates was lower but clearly feeding was massively decreased.

As a final note, this protocol presents a “golden standard” method for the assessment of feeding in daphnids which in contrast with other methods relies on small volumes, algae as food instead of any other particle and short incubation periods. Although other methods quantify algae consumption, such as with radiolabelled algae or counters which make the measurements more tedious, this protocol relies on the fluorescence of chlorophyl, which reflects the algae. Measurements in the plates are much faster and reproducible, and the required instrumentation is available in most laboratories.

## 5. Reagent Setup

***Daphnia* media:** This media is suitable for growing daphnids and is used for exposure to chemicals and incubation for feeding, based on Klüttgen et al. 1994 [20].

**Algae suspension:** The suspension of algae was prepared freshly in the culture media for daphnids at concentration ranges of 125,000 and 175,000 cells/mL. For our experiments we used a CASY counter to measure the cells and converted this to fluorescence measured with the microplate reader.

## Figures and Tables

**Table 1:** Troubleshooting and caveats.

Problem	Possible Reason	Solution
There was no difference detected between the fluorescence of wells without daphnids and the wells with daphnids for the unexposed (control) daphnids. Ideally, the unexposed animals should feed on algae and decrease their amount in the incubation period of 30 min by at least 50% from the wells without daphnids. This is actually a confirmation that the daphnids do feed on the algae during the incubation.	There are too many algae in the wells and, therefore, daphnids cannot achieve a measurable decrease by feeding. The time of the feeding incubation is too short for the daphnids to feed on the algae and make a measurable decrease in their fluorescence. There are too few animals to cause a decrease in algae.	Adjust the algae suspension to a more dilute or a lower concentration of algae in wells. This will allow the daphnids to make a measurable decrease. Adjust and increase the incubation period for feeding, i.e., if more time is required to show difference from the wells without daphnids, increase the feeding incubation to one hour. The more animals used per well, the greater the expected consumption of algae. Therefore, although the optimal number was eight or ten neonates, adjustments could be made.
The decrease in fluorescence between the unexposed (control) daphnids and those exposed to chemicals is quite similar. Therefore, there seems to be no difference in feeding between the exposed daphnids and the unexposed ones.	The chemical or the concentration of the chemical used during the exposure was too low to induce any physiological changes.	Overall, it is expected that feeding will be decreased for most exposures to chemicals, and therefore daphnids exposed to pollutants will feed less in comparison to control (unexposed) daphnids. To achieve physiological differences, increase the duration of exposure to the chemicals tested or increase the concentration of the chemical at exposure. Usually, the highest concentration to start with in the feeding assay could be at or just below the EC_20_ of the acute effects.

## Data Availability

All data for this study are provided in the Supplementary Materials as worked out examples for the application of the protocol.

## Supplementary Materials

The following supporting information can be downloaded at https://www.mdpi.com/article/10.3390/mps9040102/s1. The supplementary dataset is provided as a worked-out example with all calculations automated for the protocol presented with standard curves and differences calculated automatically.
